# ENOVAT: the European Network for Optimization of Veterinary Antimicrobial Treatment

**DOI:** 10.12688/openreseurope.18016.1

**Published:** 2024-08-07

**Authors:** Peter Damborg, Fergus Allerton, Alain Bousquet-Mélou, Chantal Britt, Petra Cagnardi, Luis Pedro Carmo, Iskra Cvetkovikj, Marcel Erhard, Annet Heuvelink, Lisbeth Rem Jessen, Gudrun Overesch, Ludovic Pelligand, Jonathan Gómez Raja, Karolina Scahill, Dorina Timofte, Ana P Vale, Kees Veldman, Els M Broens

**Affiliations:** 1 Department of Veterinary and Animal Sciences, University of Copenhagen, Frederiksberg, 1870, Denmark; 2 Willows Veterinary Centre and Referral Service, Solihull, B90 4NH, UK; 3 INTHERES, University of Toulouse, Toulouse, 31076, France; 4 School of Health Professions, Bern University of Applied Sciences, Bern, 3008, Switzerland; 5 Department of Veterinary Medicine and Animal Sciences, Universita degli Studi di Milano, Milan, Lombardy, 26900, Italy; 6 Norwegian Veterinary Institute, Ås, 1443, Norway; 7 Faculty of Veterinary Medicine-Skopje, Ss Cyril and Methodius University in Skopje, Skopje, 1000, North Macedonia; 8 RIPAC-LABOR GmbH, Potsdam, 14476, Germany; 9 Royal GD, Deventer, 7418, The Netherlands; 10 Department of Veterinary Clinical Sciences, University of Copenhagen, Frederiksberg, 1870, Denmark; 11 Institute of Veterinary Bacteriology, University of Bern, Bern, 3012, Switzerland; 12 Department of Comparative Biomedical Sciences and Department of Clinical Services and Sciences, The Royal Veterinary College, Hatfield, AL9 7TA, UK; 13 FUNDESALUD, Government of Extremadura, Mérida, 06800, Spain; 14 Infection Medicine, University of Edinburgh, Edinburgh, EH16 4 SB, UK; 15 Evidensia Södra Djursjukhuset Kungens Kurva, Kungens Kurva, 14175, Sweden; 16 Department of Veterinary Anatomy, Physiology and Pathology, University of Liverpool, Liverpool, England, CH64 7TE, UK; 17 School of Veterinary Medicine, University College Dublin, Dublin, Leinster, Dublin 4, Ireland; 18 National Reference Laboratory on Antimicrobial Resistance in animals, Wageningen University and Research, Lelystad, 8221 RA, The Netherlands; 19 Department of Biomolecular Health Sciences, Utrecht University, Utrecht, 3584 CL, The Netherlands

**Keywords:** Antimicrobial, antimicrobial resistance, antimicrobial treatment, treatment guideline, ECOFF, MALDI-TOF MS, clinical breakpoint, veterinary medicine, diagnostics, microbiology

## Abstract

The global antimicrobial resistance crisis has been the driver of several international strategies on antimicrobial stewardship. For their implementation on field level, the veterinary sector encounters several specific challenges and in particular: (i) a shortage of experts in key disciplines related to antimicrobial stewardship, (ii) a lack of evidence-based antimicrobial treatment guidelines, and (iii) inferior diagnostic tests available compared to human medicine. The present white paper describes how the COST Action ENOVAT (the European Network for Optimization of Veterinary Antimicrobial Treatment, CA18217), comprising 332 persons from 51 countries, worked towards solutions to these challenges. Initially, surveys were conducted to explore the present state in Europe in terms of existing antimicrobial use guidelines and microbiology practices performed. Concurrently, various research activities were launched to optimize diagnostics, including development of epidemiological cut-offs, clinical breakpoints and matrix-assisted laser desorption ionization time of flight mass spectrometry interpretive criteria. Also, guidelines drafting groups working towards evidence-based antimicrobial treatment guidelines for six conditions in food-producing and companion animals were established. The processes and outcomes, also in terms of capacity building, are summarized in this white paper where emphasis is placed on sustainability of the activities. Although several ENOVAT initiatives and spin-off projects will continue beyond the Action, we recommend that a new European veterinary research agenda is launched focusing on research and funding leading to long-term impacts on veterinary antimicrobial use.

## Plain language summary

Antimicrobial resistance is an urgent global public health threat that is amplified by over- and misuse of antimicrobials. As a result of antimicrobial resistance, antibiotics and other antimicrobial medicines become ineffective and infections become difficult or impossible to treat. This goes for human infections, but also for infections in animals. In a recently finished European project called ENOVAT we tried to tackle the problem of antimicrobial resistance in animals. We focused on two topics. First we optimized and harmonized diagnostics of bacterial infections in the laboratory, and second we developed evidence-based treatment guidelines to support veterinary practitioners on how and when to use antibiotics in the best way. Improved diagnostics and new treatment guidelines can help veterinary practitioners to a more sensible antibiotic choice and with that less over- and misuse of antimicrobials. This article summarizes the process and progress of the work done in the ENOVAT project. Emphasis is also put on how the project benefitted from a unique consortium encompassing 332 professionals with diverse backgrounds, from 51 countries.

## Background and aim of ENOVAT

Antimicrobial resistance (AMR) is a massive global health problem, which was estimated to contribute to 1.27 million human deaths in 2019 ( Murray *et al.*, 2022). AMR can also affect animal health and welfare, but the magnitude of the problem has not been quantified to the same extent in the veterinary setting. An additional concern with AMR in animals is the risk of zoonotic transmission of resistant pathogens or their resistance determinants through ingestion of food or via direct or indirect contact with animals ( Damborg *et al.*, 2016; Tang *et al.*, 2017).

One way to prevent the emergence and spread of AMR is through antimicrobial stewardship, which can be defined as a broad, multifaceted approach encompassing infection prevention and control measures to maximize clinical efficacy of antimicrobials while minimizing the development of AMR ( Lloyd & Page, 2018). Although this is the goal in both the human and animal sectors, veterinary medicine is faced with some specific challenges that must be overcome to facilitate proper implementation of antimicrobial stewardship. Examples of these challenges are:

1. A relative shortage of veterinary experts in key disciplines related to antimicrobial stewardship, including clinical infectious diseases, pharmacology, microbiology and infection control;
2. A lack of evidence-based veterinary antimicrobial treatment guidelines;
3. A shortage of animal- and pathogen-specific diagnostic tests and interpretive criteria;
4. A lack of harmonization in microbiological diagnostic procedures.

**Figure 1.**
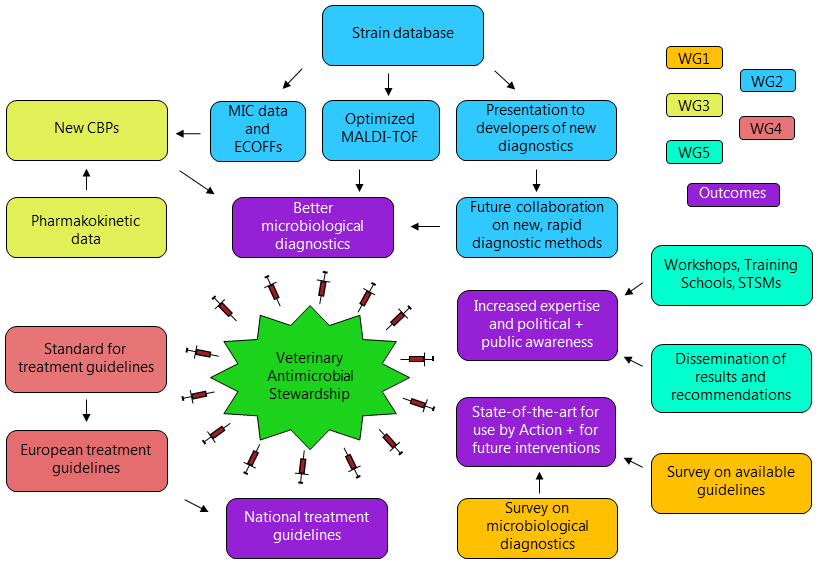
PERT chart summarizing the activities of the five working groups (WGs) within ENOVAT. CBP, clinical breakpoint; ECOFF, epidemiological cutoff; MIC, minimum inhibitory concentration; MALDI-TOF, matrix-assisted laser desorption ionization time-of-flight.

In order to meet these particular issues, members of the European Committee on Antimicrobial Susceptibility Testing (EUCAST) subcommittee of veterinary antimicrobial susceptibility testing (VetCAST) and the ESCMID study group for veterinary microbiology (ESGVM) applied for, and were granted in 2019, a COST Action entitled European Network for Optimization of Veterinary Antimicrobial Treatment (ENOVAT) ( https://cost.eu/actions/CA18217/ and https://www.enovat.eu/). **The primary aim of ENOVAT** was to optimize veterinary antimicrobial use through i) the development of animal- and disease-specific antimicrobial treatment guidelines, and ii) refinement and harmonization of microbiological diagnostic procedures. Throughout the Action, funded by COST until May 2024, 332 persons from 51 countries in Europe and beyond have been affiliated. The work of ENOVAT was performed within and across five different Working Groups (WGs) focusing on different tasks, which are outlined in Figure 1.

In this white paper, we initially summarize the specific objectives and work conducted by WGs 1–4 over the grant period. This is followed by a general discussion on i) how the magnitude and composition of this network have influenced the process and outcome, ii) how capacity building was achieved, and iii) future perspectives including continuation of activities, creation of spin-off projects, and how the overall ENOVAT network can remain sustainable. Finally, the need for a new European veterinary research agenda is discussed.

## Summary of working groups 1–4

### WG1 – mapping microbiological diagnostics and treatment guidelines

WG1 focused on two objectives: i) to describe, compare, and review the methodologies and interpretive criteria used by diagnostic laboratories across Europe for identification and antimicrobial susceptibility testing (AST) of veterinary pathogens, and ii) to map and compare the availability, structure, and evidence-base of veterinary antimicrobial treatment guidelines in Europe.

To introduce the first objective, a position paper on the need for laboratory harmonization through standardization of bacterial culture and AST was published ( Timofte *et al.*, 2021). Next, a survey on microbiological diagnostic procedures was created and disseminated to veterinary microbiological diagnostic laboratories (VMDLs) across Europe. This survey elicited responses from 290 private and public VMDLs in 34 European countries and identified a broad variety of methodologies used for bacterial culture and AST. One of the most important findings in relation to AMR surveillance was that only 48% and 46% of VMDLs routinely screened bacterial isolates for methicillin resistance and production of extended-spectrum beta-lactamase, respectively (unpublished work by Koritnik T, Cvetkovikj I, Zendri F, Blum S, Chaintoutis SC, Kopp PA, Hare C, Štritof Z, Kittl S, Goncalves J, Zdovc I, Paulshus E, Laconi A, Singleton D, Allerton F, Broens EM, Damborg P, and Timofte D). Moreover, substantial variations in the professional qualifications of staff, use of quality assurance, diagnostic procedures for bacterial culture and identification, methods and standards used for performing and interpreting AST, and reporting of results, were observed among survey respondents. The findings from the survey reinforces the need for greater harmonization of bacteriology methodologies. Therefore, a core group of ENOVAT participants was subsequently established aiming to create harmonized protocols for veterinary microbiology investigations. This initiative is further addressed in the discussion.

For the second objective, existing national antimicrobial use guidelines for companion animals in Europe were initially mapped via a thorough search of national resources assisted by representatives from all ENOVAT member countries ( Allerton *et al.*, 2021). Only fifteen different resources were identified from 11 of the 40 countries surveyed, highlighting an important gap in national guidelines for companion animals. The study used the Appraisal of Guidelines for Research and Evaluation (AGREE II) framework to evaluate the guidelines on the following parameters: scope & purpose, stakeholder involvement, rigour of development, clarity and presentation, applicability and editorial independence ( Brouwers *et al.*, 2016). One important outcome of the AGREE II analysis was a general failure to report the methodological steps undertaken to evaluate available evidence and to formulate recommendations. This result likely reflects a general lack of published evidence and that treatment guidelines are often based on expert consensus. It is hoped that an improved understanding of the limitations of existing resources can support guideline developers to optimize future iterations to meet their stewardship objectives. A subsequent treatment guidelines survey was designed and translated by ENOVAT country representatives into 27 languages to determine awareness of these existing antimicrobial use guidelines among European veterinary practitioners, and also stakeholder preferences as to the format and content of future tools. The survey obtained answers from 2,271 companion animal practitioners from 46 different countries and identified a correlation between a surrogate measure of optimal antimicrobial usage and awareness of antimicrobial stewardship guidelines ( Farrell *et al.*, 2024). Such awareness was greatest in countries that have their own national guidelines. Consequently, national bodies are strongly encouraged to create new, or adapt existing, resources to improve local dissemination. The survey also identified key features, including preferences for a web-based interface and inclusion of agent selection, dosing and treatment duration information that should be incorporated in future guideline documents to improve their uptake. Later in the Action, a similar survey was sent to equine practitioners across Europe. The results of that survey are pending.

The outcomes of WG1 tasks can benefit veterinary diagnosticians worldwide. Following our newly attained understanding of the diversity of microbiology laboratory practices, the development of international guidelines for laboratory processing of veterinary clinical specimens is expected to gradually harmonize laboratory practices and thereby positively impact guidance around antibiotic selection. Furthermore, by understanding the preferences of key stakeholders regarding the format and content of antimicrobial use guidelines, developers can prepare future resources that are optimized for veterinary practitioners – improving adherence and rational antimicrobial use.

### WG2 – creating a European strain database, ECOFFs and MALDI-TOF MS criteria

The objectives of WG2 were to establish a strain database with information on animal bacterial pathogens stored across European laboratories, and to use these strains for:

1. Refining identification procedures for veterinary pathogens by matrix-assisted laser desorption ionization time of flight mass spectrometry (MALDI-TOF MS).
2. Production of aggregated minimum inhibitory concentration (MIC) distributions for determination of epidemiological cut-offs (ECOFFs).

First, WG2 agreed by consensus on bacterial species to be included in the strain database. Since ECOFFs can be used for AMR monitoring and constitute a prerequisite for setting clinical breakpoints (CBPs, see WG3), the focus was on veterinary-relevant bacterial species lacking ECOFFs for clinically relevant antimicrobials. Furthermore, common animal pathogens of high clinical and/or economic importance and known to be difficult to identify by MALDI-TOF MS, were selected. To create the strain database, a survey with the bacterial species wish list was created and sent to private and public diagnostic laboratories across Europe in which participants were asked to share information on their strains including relevant metadata. This resulted in a database currently (August 2024) comprising detailed data on more than 26,000 bacterial isolates stored in laboratories located in 24 countries. A report summarizing the current content of the database has been published on the ENOVAT homepage ( https://enovat.eu).

To refine MALDI-TOF MS bacterial identification, a general step-by-step guideline was developed. By applying this guideline for *Staphylococcus* ( *S.*) *intermedius* group isolates from the database, combinations of spectral masses specific for selected species within this group could be identified. The guideline, which is yet to be published, has also been used for other selected species/genera ( *Streptococcus* ( *S.*) *canis*, *S. dysgalactiae* subspecies, *S. porcinus* and *S. equi* subspecies, *Mycoplasmopsis*, *Mycoplasmoides*, *Mesomycoplasma* and *Metamycoplasma* species, *Campylobacter* ( *C.*) *hepaticus* and *C. bilis*, the *Aeromonas salmonicida* group, and *Actinobacillus* species). Thereby, it turned out that the underlying difficulties for reliable identification by MALDI TOF MS are diverse depending on the bacterial species in question. For *C. hepaticus* and *C. bilis*, as well as for nine species from the *Mycoplasmopsis*, *Mycoplasmoides*, *Mesomycoplasma* and *Metamycoplasma* groups, the lack of reliable mass spectra in the commercial MALDI-TOF MS databases was the underlying problem. Work on remaining bacterial species is ongoing, but it has proven nearly impossible to develop MALDI-TOF MS criteria for certain *Aeromonas* species due to identical 16S sequences and the lack of a reproducible gold standard for their identification to the species level. Even though the strain database showed its potential as basis for MALDI-TOF MS optimization, a database including reliable mass spectra from well-identified organisms can be more feasible for this purpose in a longer term. An example of such a database is the open access MALDI-UP Catalogue ( https://maldi-up.ua-bw.de/catalogue.asp), designed and curated by the MALDI-UP User Platform ( https://maldi-up.ua-bw.de), which is intended for exchange of local mass spectra between MALDI-TOF MS databases of different laboratories. WG2 has established collaboration with the developers of the MALDI-UP Platform to facilitate further work on bacterial species identification beyond the project period. Moreover, preliminary studies with a commercially available MALDI⁠⁠⁠⁠⁠⁠⁠⁠⁠⁠⁠⁠⁠⁠⁠⁠⁠⁠⁠-⁠⁠⁠⁠⁠⁠⁠⁠⁠⁠⁠⁠⁠⁠⁠⁠⁠⁠⁠TOF MS database ( https://mabritechcentral.com) using marker masses from whole genome sequence data showed promising results for identification when uploading mass spectra of bacterial species without reliable identification.

The selection of pathogen/antimicrobial combinations for ECOFF determination was done in close collaboration with WG3 to account for the CBPs prioritized by this group. It was decided to focus on eight first-line penicillins and tetracyclines and six bacterial species, namely *Staphylococcus aureus*, *Staphylococcus pseudintermedius*, *S. equi* subsp. *equi* and *zooepidemicus*, *Actinobacillus pleuropneumoniae*, *Pasteurella multocida*, and *Mannheimia haemolytica*. The work to generate MIC distributions involved five laboratories from the ENOVAT consortium, 1,000 custom-made commercial broth microdilution plates (Sensititre, Thermo Fisher Scientific), and 20 isolates per bacterial species, obtained from the above-mentioned European strain database and other strain collections. Susceptibility testing was performed with broth microdilution according to EUCAST standards. After generation of MIC distributions, these were reviewed and aggregated to determine new ECOFFs according to EUCAST SOP 10.2 ( Anonymous, 2021). As a result, 15 new ECOFFs and seven new tentative ECOFFs were set by the EUCAST steering committee. Some challenges were encountered, e.g. truncated MIC distributions and unexpected tetracycline MIC differences between the two *S. equi* subspecies included. Therefore, further work is needed to solve these issues. Apart from (T)ECOFFs, new quality control test ranges for the reference strains *Staphylococcus aureus* ATCC 29213 and *Streptococcus pneumoniae* ATCC 49619 were developed for several antimicrobials. These quality control test ranges and the newly generated (T)ECOFFs are publicly available on the EUCAST homepage ( www.eucast.org).

### WG3 – developing clinical breakpoints

The objectives of WG3 were i) to make a priority list of animal- and infection-specific CBPs that are currently lacking for animal pathogens, and ii) to develop CBPs for major animal species using ECOFFs and pharmacokinetic/pharmacodynamic (PK/PD) cut-offs.

The priority list of CBPs was developed by consensus within WG3 and based on identification of important gaps in existing internationally recognized breakpoint tables for animal pathogens. Additional considerations favoring selection of breakpoints included a relatively high level of consumption of antibiotics for the infection, prioritization of the infection for guideline development by WG4, and availability of PK data (including plasma protein binding) and PD data such as MIC distributions and time-kill kinetics. Only infections requiring systemic antibiotic use were considered, and infections for both food-producing and companion animals were included in the list. Moreover, ENOVAT members were asked by a survey which CBPs were most urgently needed, and 137 responses were received. Most respondents were microbiologists (50%), followed by pharmacologists (15%) and clinicians in small (15%) or large (15%) animal practice. According to survey results, CBPs for sulphonamide/trimethoprim combinations for dogs, horses, swine and cattle were of the highest priority. This is understandable, since currently no single animal-specific sulphonamide/trimethoprim CBP exists. However, defining a CBP for drug combinations like sulphonamide/trimethoprim is challenging because a variety of sulphonamide/trimethoprim combinations with different pharmacokinetic properties and variable synergism between the two components are available in the EU. The MIC definition of drug combinations is also complicated, as different ratios of the compounds can be tested. Considering these issues, the sulphonamide/trimethoprim combination was not included in the priority list, but it will be the objective of future studies.

This selection process resulted in the following list of prioritized CBPs:

1. Amoxicillin-clavulanic acid in dogs administered IV and PO against soft tissue infections caused by *S. pseudintermedius*, *S. aureus*, *P. multocida*, Enterobacterales and *Enterococcus* spp. (supported by Vegas *et al.*, 2021).
2. Penicillin procaine and penethamate in horses against *Staphylococcus* spp., *S. equi* subsp. *equi* and *zooepidemicus* infections (supported by Lallemand *et al.*, 2023).
3. Oxytetracycline in cattle against *M. haemolytica* and *P. multocida* infections.
4. Doxycycline in pigs against *A. pleuropneumoniae* and *P. multocida* infections.
5. Doxycycline in poultry against Avian Pathogenic *E. coli* (APEC).

CBP determination was done according to the process described by Toutain *et al.* (2017). Briefly, it comprises the determination of two or three critical MICs needed to assist in the selection of the CBP. These MIC cut-off values are i) the ECOFF, (ii) a PK/PD cut-off obtained from pre-clinical and clinical pharmacokinetic raw data, which is the highest possible MIC for which a given percentage of animals in the target population achieves a critical value for the selected PK/PD index (fAUC/MIC or fT>MIC), and (iii) when possible, a clinical cut-off, which could be obtained by analyzing the relationship between MIC values and clinical cure. WG2 was the main contributor of ECOFFs, whereas other PD data (e.g. time-kill data) and PK data were obtained from literature searches, requests to pharmaceutical industry and academic collaborators, and *in vitro* and *in vivo* studies conducted by research groups affiliated to the EUCAST subcommittee VetCAST. Mathematical modelling was then performed on collected PK and PD data, and – when available – clinical efficacy data were incorporated for the creation of veterinary-specific rationale documents that will inform the CBPs. So far, 10 CBPs have been proposed for amoxicillin-clavulanic acid in dogs (n=5) and for benzylpenicillin procaine in horses (n=2), and for oxytetracycline in cattle (n=3). At the present time (August, 2024), these are available in 3 rationale documents in consultation on the EUCAST website.

These CBPs will be published in dedicated breakpoint documents that VetCAST is preparing, in line with the EUCAST breakpoint documents. CBPs for doxycycline in poultry and pigs and sulphonamide-trimethoprim will be addressed in the near future as a continuation of ENOVAT.

### WG4 – developing of evidence-based treatment guidelines

The overall aim of WG4 was to develop antimicrobial use guidelines to help veterinarians optimize antimicrobial use and improve animal care. To achieve this goal, WG4 focused on three objectives: (i) to draft a standard for evidence-based veterinary clinical guidelines; (ii) to write European evidence-based veterinary clinical guidelines for antimicrobial use in a number of prioritized conditions in food-producing and companion animals and (iii) to promote the transformation of ENOVAT guidelines into national/regional guidelines in Europe. A secondary aim of the guidelines’ initiative was to build veterinary capacity within guidelines methodology.

When phrasing the standard for veterinary clinical guidelines, the working group focused on end-user and stakeholder involvement and the application of a systematic and transparent assessment of supporting evidence. For this purpose, the ENOVAT operating procedure (OP) describes adherence to the AGREE II framework for guidelines ( Brouwers *et al.*, 2016) and the Grading of Recommendations Assessment, Development and Evaluation (GRADE) approach ( Guyatt *et al.*, 2008a; Guyatt *et al.*, 2008b). The GRADE approach relies on transparent and systematic search for evidence and rating of the certainty of the evidence. The disease conditions prioritized for guideline development were selected by consensus based on the amount and critical importance of the antimicrobials used for treatment of these conditions, the potential of guidelines to impact animal and public health, and lack of similar European guidelines. For each condition selected, drafting groups comprising various experts from the ENOVAT network, in particular clinical experts, field clinicians, methodologists, microbiologists, and pharmacologists, were established. To the extent possible, the broad geographical coverage of ENOVAT was exploited to ensure members from different countries were included in each drafting group. The following six conditions were selected for guidelines development by WG4 drafting groups: colibacillosis in poultry, bovine mastitis, bovine respiratory disease, post-weaning diarrhoea in pigs, canine acute diarrhoea, and surgical prophylaxis in companion animals.

Briefly, drafting group members developed the most relevant questions to be addressed by the guidelines. All treatment questions were phrased using the Population Intervention Comparator Outcome (PICO) framework and informed the literature search. To that end a literature review protocol was developed and deposited in SYREAF (online platform for Systematic Reviews for Animals and Food). For the search strategy, several bibliographic databases using different interfaces were used and all studies identified were exported to a review manager software. Upon reaching consensus between two independent drafting group members on eligible abstracts, manuscripts were subjected to full-text screening. Data from all included papers were inserted in a data management software followed by meta-analysis. The contextualized GRADE methodology was applied to evaluate the certainty of evidence. Guideline recommendations were drafted by the drafting groups during a face-to-face/hybrid meeting and informed by data retrieved from the systematic review, stakeholder interviews and any other relevant information. Drafting of recommendations followed the GRADE Evidence to Decision (EtD) framework, taking into consideration the certainty of the evidence, the balance between benefits and harms and values and preferences of end-users. The final step of guideline development was the public consultation phase.

Until now, one scoping and two systematic reviews ( Paudel *et al.*, 2024; Scahill *et al.*, 2024; Soerensen *et al.*, 2024), and several other papers supporting decision making ( Kortstegge *et al.*, 2023; Pelligand *et al.*, 2024; Preine *et al.*, 2022) have been published by the drafting groups. In addition, one treatment guideline on canine acute diarrhoea has been submitted for publication, and one on surgical prophylaxis in dogs and cats is in preparation for public consultation. The remaining work will become available after ENOVAT terminates. During the past three years, results of the evidence synthesis and/or the derived guideline recommendations have been disseminated widely at conferences, meetings and at webinars in Europe and beyond. While international dissemination has been successful for much of the work, the transformation of ENOVAT guidelines into national guidelines is an objective yet to be achieved.

ENOVAT guidelines will be the first evidence-based antimicrobial use guidelines developed for the international veterinary community. The process has been monitored by the working group leadership and drafting group members have participated in surveys to document challenges and facilitators of the process. Results of this evaluation will be made available in a separate research publication. During the course of the Action, the ENOVAT network has reached out and attracted methodologists from the human medical field. Several training activities in evidence synthesis and guidelines methodology have been conducted with teaching by methodologists from the GRADE expert group and the European Society for Clinical Microbiology and Infectious Disease (ESCMID).

## Discussion of ENOVAT collaboration, capacity building and sustainability

Overall, ENOVAT activities have contributed to strengthening inter- and transdisciplinary collaboration between animal health professionals based in different European countries. This collaboration was a true benefit for the individual WGs, as expertise at many levels contributed to fulfilling the objectives of the Action. For instance, collaboration was essential to achieve the WG3 objectives of defining new CBPs, as this process depends on microbiologists to determine ECOFFs, pharmacologists to conduct PK/PD modelling and clinicians to help determine the clinical relevance of proposed CBPs. Another example is WG4, which benefitted not only from veterinary practitioners and their expertise from a clinical perspective, but also methodologists trained in the GRADE and AGREE 2 approaches, as well as microbiologists providing input on condition-specific pathogens and their resistance profiles.

Besides expertise, the ENOVAT network also had the advantage of bringing together participants from different countries covering most of Europe and some countries outside Europe. ENOVAT country representatives were able to translate and disseminate surveys with the support of national stakeholders and agencies, as well as governmental and private diagnostic laboratories. Linguistic support was also provided to generate multiple versions of an educational animation (see: ENOVAT videos on the rational use of antibiotics – ENOVAT) produced to convey key stewardship information to pet owners ( Wright *et al.*, 2024). These national networks will also be valuable beyond the completion of ENOVAT, for the translation, promotion and implementation of current and future ENOVAT treatment and laboratory procedure guidelines, CBPs and other outcomes. This is particularly important for countries and regions that are often underrepresented in stewardship initiatives. At a higher level, international organizations such as EFSA, ESCMID, FAO, and WOAH (all represented in the ENOVAT advisory board) may also contribute with their strong voices and wide reach to help the dissemination of ENOVAT outcomes within and beyond Europe.

One very important aspect of ENOVAT was capacity building. Even if this is difficult to measure quantitatively, we are confident that the critical mass of European expertise in veterinary microbiology, pharmacology, internal medicine, epidemiology and more broadly in veterinary antimicrobial stewardship was expanded during the lifetime of ENOVAT. A particular focus was on the involvement of young researchers and members from Inclusiveness Target Countries (ITCs) in all working group activities. Furthermore, a total of seven training schools were held, including three concerning PK/PD principles and breakpoint-setting in veterinary pharmacology, two on diagnostic microbiology, and two on creation of evidence-based treatment guidelines. On top of that, 20 short-term scientific missions and 16 virtual mobility grants were completed with physical and online research exchanges, respectively. Examples of tasks performed during these exchanges include building surveys and analyzing data produced during the Action. In addition, these visits were used to expand the network for early-stage researchers, and for them to learn methods such as diagnostic laboratory approaches or PK/PD modelling. One specific example of successful involvement of ITCs is the organization of the international conference *“Antimicrobial Resistance in Veterinary Medicine – Current State and Perspectives”* in Novi Sad, Serbia (ISBN: 978-86-7520-555-5). After a successful first edition in 2022 with several keynote speakers and participants supported by ENOVAT grants, a second and third edition took place in 2023 and 2024 – the latter after the termination of ENOVAT. This annual conference attracts hundreds of participants, mainly from South and East-Europe (Balkans), enabling knowledge transfer and capacity building in these regions.

Many communication and dissemination activities were performed to ensure that findings are shared with stakeholders, the scientific community, healthcare professionals, policymakers, and the public. Several promotional and educational videos were produced, and disseminated through the different social media channels and the website.

It is important to emphasize that the termination of the ENOVAT project as a COST Action by May 2024 does not mean the end of the research and other initiatives launched. Apart from the above-mentioned conference in Serbia, a few examples are highlighted in the following:

1. The comprehensive work towards treatment guidelines will continue for the WG4 drafting groups that have multiple ongoing projects. In extension to this, ENOVAT affiliates have established a veterinary project group together with methodologists from the official GRADE working group ( https://www.gradeworkinggroup.org/). An extension of this work towards a veterinary-specific GRADE approach and the education of veterinary methodologists to become proficient in its application will undoubtedly benefit and promote the future development of additional evidence-based veterinary antimicrobial treatment guidelines.
2. An initiative arising from ENOVAT is the Companion Animal Microbiology Protocols (CAMiProt). The CAMiProt resource is a voluntary initiative by a core group of microbiologists, based on the WG1 survey revealing major inconsistencies in the diagnostic microbiology procedures performed in veterinary laboratories across Europe. The objective is to harmonize the diagnostic approach for bacteriological diagnostic procedures applied to clinical samples from companion animal infections. Samples from other animal species might be included at a later stage. To ensure the sustainability and updates of this archive, it will be adopted and hosted by the European College of Veterinary Microbiology.
3. ENOVAT’s work towards additional ECOFFs and CBPs, including priority CBPs from the WG3 survey will continue under the umbrella of the EUCAST subcommittee VetCAST.
4. 4. The novel WG2 strain database will remain available, and could possibly be extended, as a valuable toolbox to support future diagnostic research beyond the continued development of ECOFFs and new MALDI-TOF MS interpretive criteria.

Besides these tangible extensions of ENOVAT, several project affiliates have identified new collaborators in the consortium, within and across countries and research fields. It should be mentioned that, at the end of the Action, uncertainty pertains to the name “ENOVAT” and its future platform. Opportunities are therefore being explored for ENOVAT to continue under the umbrella of an existing, related organization, or as an independent association.

## A new European veterinary research agenda?

One of the objectives of ENOVAT was to outline how European countries may advance to a common high level of veterinary antimicrobial stewardship. This is a complex task requiring investments beyond networking projects like ENOVAT. Importantly, the European Commission (EC) encourages member states to regularly update and implement National Action Plans (NAPs) against AMR in humans, animals, and the environment. For this purpose, a One Health approach is needed, however the starting point varies between the different sectors of One Health. In human medicine, research, surveillance and education on many aspects of antimicrobial stewardship started decades ago leading to awareness, evidence-based guidelines and effective AMR intervention strategies. On the other hand, ENOVAT has underlined that scientific evidence is lacking to reach similar goals for veterinary medicine in the short term. This knowledge, and lessons learned in human medicine, can be used by the EC for the establishment of a new European veterinary research agenda. One example of what to include is research into education that will impact antibiotic usage patterns in different animal sectors. In that regard, it is imperative to identify educational initiatives with high impact in different countries having different prerequisites, culture and traditions for antibiotic use. In view of ENOVAT results, research into microbiological diagnostics and its role in driving antimicrobial use and stewardship, would also fit well in a European research agenda. For this topic, it should be acknowledged that resources vary between countries and that simple and cheap solutions, ideally at the point-of-care, may have a bigger impact in some countries as opposed to state-of-the-art diagnostics requiring expensive equipment. Finally, realizing that the creation of evidence-based antimicrobial treatment guidelines depends on – largely non-existing - *evidence*, the agenda should acknowledge the urgent need for randomized controlled treatment studies in animals with different infections. Ideally, any research conducted under this new agenda should be followed by investments to implement solutions found to impact veterinary antimicrobial stewardship.

With the recent experience of ENOVAT, we have learned the value of bringing together experts from different scientific fields. We therefore hope that our experience can serve as an inspiration for the EC to take antimicrobial stewardship one step further, so that not only ENOVAT but the entire topic of “veterinary antimicrobial stewardship” becomes sustainable and prioritized in the years to come.

## Conclusion

Over 4.5 years, ENOVAT has completed nearly all originally scheduled tasks related to the development of treatment guidelines and refinement of microbiological diagnostics in the veterinary setting. Also, capacity in important veterinary fields related to antimicrobial stewardship has been built across Europe. The actual impact of these initiatives on veterinary antimicrobial usage remains to be assessed, but the potential exists for e.g. international evidence-based treatment guidelines to result in paradigm shifts for treatment of certain animal infections – not only in Europe but at a larger international scale. In terms of sustainability, several new research collaborations, sub-projects as well as spin-off initiatives will continue beyond ENOVAT. Ultimately, the authors hope that the ENOVAT brand and work will inspire the creation of a new European veterinary research agenda aiming towards long-term solutions within veterinary antimicrobial stewardship.

## Disclaimer

The views expressed in this article are those of the author(s). Publication in Open Research Europe does not imply endorsement of the European Commission.

## Ethics and consent statement

This article summarizes results obtained in the COST Action ENOVAT. Ethical approval and consent were not required for this article. When necessary for individual studies and survey, ethical approval was received

## Data Availability

This article summarizes results obtained in the COST Action ENOVAT. No actual data are associated with this article. Detailed research results, including data, will be published separately.
