# Supplementary material for: ENOVAT: the European Network for Optimization of Veterinary Antimicrobial Treatment
Source: Open Res Eur. 2024 Sep 24;4:170. Originally published 2024 Aug 7. [Version 2] doi: 10.12688/openreseurope.18016.2 (PMC11380070; doi:10.12688/openreseurope.18016.2)
Supplement: Peer review history

# Reviewer report: Reviewer response for version 2

**DOI:** 10.21956/openreseurope.20088.r44557

**Published:** 2024-09-25

**Authors:** Ilias Chantziaras

**Affiliations:** 1 University of Ghent, Ghent, Belgium

No further comments to make.

Where applicable, are recommendations and next steps explained clearly for others to follow? (Please consider whether others in the research community would be able to implement guidelines or recommendations and/or constructively engage in the debate)

Yes

Does the article adequately reference differing views and opinions?

Yes

Are all factual statements correct, and are statements and arguments made adequately supported by citations?

Yes

Is the rationale for the Open Letter provided in sufficient detail? (Please consider whether existing challenges in the field are outlined clearly and whether the purpose of the letter is explained)

Yes

Is the Open Letter written in accessible language? (Please consider whether all subject-specific terms, concepts and abbreviations are explained)

Yes

Reviewer Expertise:

one health, biosecurity, veterinary epidemiology, animal health management

I confirm that I have read this submission and believe that I have an appropriate level of expertise to confirm that it is of an acceptable scientific standard.

# Reviewer report: Reviewer response for version 1

**DOI:** 10.21956/openreseurope.19471.r43009

**Published:** 2024-09-06

**Authors:** Ilias Chantziaras

**Affiliations:** 1 University of Ghent, Ghent, Belgium

The authors are to be commended on the excellent work that they have done during this COST action. There have been plenty of activities performed to explore the existing microbiology practices and any antimicrobial use guidelines performed among the participating countries. Not only that, but numerous other activities were devoted to optimise and promote improved practices and evidence-based guidelines. 

 With regards to the manuscript, it is well-written, using easy-to-follow language and providing several (some of which measurable) outcomes and key points that should help the stakeholders (e.g. policy makers) that would like to use them towards promoting the agenda of antimicrobial stewardship.

 One general comment that I would give is to structure the white paper more as a "problem-solution" style and less as a presentation of the working packages. For instance, instead of the title "summary of working groups 1-4. I would instead use a title that informs over **what did ENOVAT do** to optimize veterinary antimicrobial use and dissect all activities according to the two main aims that could be the two main subtitles of this section,**

*i) ENOVAT's work towards the development of animal- and disease-specific antimicrobial treatment guidelines*, and

*ii) ENOVAT's work towards the refinement and harmonization of microbiological diagnostic procedure*s.

 For example, in *i) ENOVAT's work towards the development of animal- and disease-specific antimicrobial treatment guidelines *we would list the activities performed in WG1 and WG4

 and for *ii) ENOVAT's work towards the refinement and harmonization of microbiological diagnostic procedures *all the activities performed in WG1-3. This way, it will facilitate the better dissemination of the great work that has been performed as there are complimentary activities that span through different WGs. 

 Regarding the title "Discussion of ENOVAT collaboration, capacity building and sustainability", I think that could be renamed with something like "Collaboration through ENOVAT: capacity building and legacy (of collaboration)". 

 Similarly, I would rename the title "A new European veterinary research agenda?" to "A new European veterinary research agenda to improve antimicrobial stewardship?" and then signify somehow (e.g. bullet points, in bold) the steps:

 - One Health approach. Ideally, I would maybe propose for systems thinking to be employed on the next steps *e.g. *for AMR the work of Rüegg *et al., 2018 [Ref-1]* and Arnold et al., 2024 [Ref - 2] and  

 -Directed research to fill in specific gaps ( *e.g. *antibiotic usage patterns education, RCTs for creation of evidence-based antimicrobial treatment guidelines)

Where applicable, are recommendations and next steps explained clearly for others to follow? (Please consider whether others in the research community would be able to implement guidelines or recommendations and/or constructively engage in the debate)

Yes

Does the article adequately reference differing views and opinions?

Yes

Are all factual statements correct, and are statements and arguments made adequately supported by citations?

Yes

Is the rationale for the Open Letter provided in sufficient detail? (Please consider whether existing challenges in the field are outlined clearly and whether the purpose of the letter is explained)

Yes

Is the Open Letter written in accessible language? (Please consider whether all subject-specific terms, concepts and abbreviations are explained)

Yes

Reviewer Expertise:

one health, biosecurity, veterinary epidemiology, animal health management

I confirm that I have read this submission and believe that I have an appropriate level of expertise to confirm that it is of an acceptable scientific standard.

## References

[1] : A Systems Approach to Evaluate One Health Initiatives. *Front Vet Sci* .2018;5: 10.3389/fvets.2018.00023 23 10.3389/fvets.2018.00023 29594154 PMC5854661

[2] : The need for One Health systems-thinking approaches to understand multiscale dissemination of antimicrobial resistance. *Lancet Planet Health* .2024;8(2) : 10.1016/S2542-5196(23)00278-4 e124-e133 10.1016/S2542-5196(23)00278-4 38331529

# Response

**Published:** 2024-09-16

**Authors:** Peter Damborg

**Affiliations:** Department of Veterinary and Animal Sciences, University of Copenhagen, Frederiksberg, Denmark

Although we understand the point raised about restructuring the paper, we deliberately chose to structure the paper in the same way as referred to in Figure 1. This illustrates how the work of this COST Action was planned and structured into working groups. We have therefore decided to keep the original text. We have updated the headings from "Discussion of ENOVAT collaboration, capacity building and sustainability" to “Collaboration through ENOVAT: capacity building and sustainability”, and from "A new European veterinary research agenda?” to “A new European veterinary research agenda to optimize veterinary antimicrobial treatment?”. We believe that our ideas for a new veterinary research agenda are in line with what is proposed by the reviewer (e.g. we have already proposed a One Health approach for updating NAPs, and we have emphasized the need for RCTs). Therefore, we have decided not to revise the paragraph on a proposed new research agenda.

# Reviewer report: Reviewer response for version 1

**DOI:** 10.21956/openreseurope.19471.r43010

**Published:** 2024-09-03

**Authors:** Kirsten E. Bailey

**Affiliations:** 1 Melbourne Veterinary School, University of Melbourne, Victoria, Australia

The open letter "ENOVAT: the European Network for Optimization of Veterinary Antimicrobial Treatment" provides a clear overview of the activities conducted by four working groups, publications reporting these results and where to find them. The process and progress achieved by the ENOVAT project are excellent and highly valuable to antimicrobial stewardship efforts within the veterinary community, not only in Europe, but globally.  

 A few minor edits are suggested below.

 In the second sentence of the abstract change "For their implementation **on** field level" to "For their implementation **at the** field level".

 In the plain language summary consider rewording "Improved diagnostics and new treatment guidelines can help veterinary practitioners **to a** more sensible antibiotic choice **and with that** less over- and misuse of antimicrobials" to "Improved diagnostics and new treatment guidelines can help veterinary practitioners **make** more sensible antibiotic choices, **resulting in** less over- and misuse of antimicrobials"

 On page 6, Edit the sentence "Even though the strain database showed its potential **as basis** for MALDI-TOF MS optimization, a database including reliable mass spectra from well-identified organisms can be more feasible for this purpose in **a longer **term." to "Even though the strain database showed its potential as **the** basis for MALDI-TOF MS optimization, a database including reliable mass spectra from well-identified organisms can be more feasible for this purpose in **the long** term."

 Also, there is no explanation for the abbreviation (T)ECOFFs. I suggest the authors to address this.

 On page 8, I believe the statement 'ENOVAT guidelines will be the first evidence-based antimicrobial use guidelines developed for the international veterinary community' may not be entirely accurate. The ISCAID guidelines, for example, are also evidence-based and developed for the international veterinary community. Could you clarify whether the distinction lies in the methodology and rigor of development, or in the specific animal species and conditions covered? It might be helpful to reword this sentence to better reflect these differences.

 On page 9 in the conclusion, consider replacing the abbreviation **e.g.** with the word **example** so the sentence would read "...but the potential exists, for **example, **for international evidence..."

Where applicable, are recommendations and next steps explained clearly for others to follow? (Please consider whether others in the research community would be able to implement guidelines or recommendations and/or constructively engage in the debate)

Yes

Does the article adequately reference differing views and opinions?

Yes

Are all factual statements correct, and are statements and arguments made adequately supported by citations?

Yes

Is the rationale for the Open Letter provided in sufficient detail? (Please consider whether existing challenges in the field are outlined clearly and whether the purpose of the letter is explained)

Yes

Is the Open Letter written in accessible language? (Please consider whether all subject-specific terms, concepts and abbreviations are explained)

Partly

Reviewer Expertise:

Veterinary microbiology, susceptibility testing, antimicrobial use and prescribing guidelines and veterinary stewardship interventions.

I confirm that I have read this submission and believe that I have an appropriate level of expertise to confirm that it is of an acceptable scientific standard.

# Response

**Published:** 2024-09-16

**Authors:** Peter Damborg

**Affiliations:** Department of Veterinary and Animal Sciences, University of Copenhagen, Frederiksberg, Denmark

We have followed all suggestions for revision of language. The term "evidence-based guidelines" implies that a rigorous methodology was applied to search and assess the available evidence. For treatment guidelines, this involves a systematic review being performed and application of a grading system for assessment of the certainty of the identified evidence. Although the current ISCAID guidelines take into consideration, and reference, the literature, they are not evidence-based. They will be in the future.
